# Setbacks in Alzheimer research demand new strategies, not surrender

**DOI:** 10.1371/journal.pmed.1002518

**Published:** 2018-02-27

**Authors:** Björn Jobke, Thomas McBride, Linda Nevin, Larry Peiperl, Amy Ross, Clare Stone, Richard Turner, as the PLOS Medicine Editors

**Affiliations:** Public Library of Science, San Francisco, California, United States of America, and Cambridge, United Kingdom

## Abstract

In this month’s editorial, the *PLOS Medicine* Editors discuss the challenges of addressing a growing population with Alzheimer disease and dementia amidst disappointing news from the pharmaceutical industry.

Those hoping for new treatments for Alzheimer disease (AD) are unfortunately used to receiving disappointing news, but this year began on a particularly sobering note when Pfizer announced that it was shutting down its drug-discovery efforts for the disease [1]. A pharma giant giving up on such a huge potential market—globally, 47 million people are affected by AD and related dementias, and the number is predicted to double every 20 years [2]—is a telling acknowledgment of how barren the AD drug-discovery landscape is.

The path to a drug that can halt AD is beset by difficulties, as several pharmaceutical companies have experienced. Trials for such drugs have, in most instances, failed—many at later stages of clinical evaluation, after considerable financial investment. For instance, Eli Lilly abandoned development of the injectable drug solanezumab, which targets β-amyloid protein but in clinical trials failed to improve cognition or function in patients with mild-to-moderate AD, for those receiving the drug compared to those on placebo [3,4]. Similarly, TauRX experienced discouraging results with their drug LMTM, which targets tau protein [5].

AD presents a unique combination of challenges to drug-discovery efforts. Treating any brain disease requires getting drugs across the blood—brain barrier and avoiding toxicity from off-target effects in the complex network of cerebral activity. Treating diseases of aging carries the added complication of managing comorbidities. In the case of AD, the extracellular deposits of amyloid-β peptide and the intracellular accumulation of abnormally phosphorylated tau neurofibrillary tangles (NFT) that define the disease are often accompanied by other neurological insults that also affect cognition, such as cerebrovascular disease, Lewy bodies, frontotemporal lobar degeneration, or hippocampal sclerosis [6–8]. It is estimated that 16% or more of people believed by their physician to have probable AD are in fact misdiagnosed and have another type of neurocerebral pathology [9]. Difficulty identifying the early stages of AD also means that many trial participants with accurately diagnosed AD are at an advanced stage of the disease, with neurological damage that may not benefit even from a drug that can effectively prevent further amyloid plaque or tangle formation.

The slow progression of AD, coupled with the costly cognitive and imaging tests used to establish clinical end points and monitor safety, makes trials lengthy and expensive. With promising results now coming from trials of cancer therapies—especially immunomodulatory approaches—one can see how pharma companies might find it more attractive to turn their attention to drugs that generally cost less to test: Phase III Alzheimer trials cost hundreds of millions of dollars to conduct, whereas a cancer trial costing closer to US$50 million may have a better chance of success. Nonetheless, the burden of AD and other forms of dementia cannot be ignored, and this area continues to be a funding priority for governments (the United States National Institutes of Health budget for AD and AD-related disease research is currently US$1.4 billion per year) and nonprofits (e.g., US$385 million from the Alzheimer’s Association International Research Grant Program). We hope that these investments will result in a better understanding of the mechanisms and biomarkers in AD, and perhaps a drug-discovery landscape with greater certainty will convince companies like Pfizer to recommit resources.

While it may be tempting to focus on results of drug trials with the implicit hope of imminent clinical benefit, meaningful success against dementia must remain a longer-term project. Bruce Miller and Carol Brayne, co-guest editors of *PLOS Medicine’s* 2017 Special Issue on Dementia, note that the focus on single biological targets to treat such a complex disease may have sown the seeds for clinical failure. “Drug development researchers have been ignoring what population data and the more detailed work from subsets of rarer dementia have been telling us for a long time,” says Brayne. Miller agrees that “targeted approaches on smaller cohorts with better understood biology will be a better approach,” noting also that “Pfizer and others took an overly simplistic approach thinking essentially that all dementia—whatever age—would respond to amyloid-lowering therapies.”

A more personalized approach to AD will require acknowledging the combinations of dementia pathologies that contribute to cognitive deficits in people with AD and treating these entities in parallel with amyloid- and NFT-directed or novel AD-specific interventions. Such an approach will likely require earlier diagnosis and more sophisticated understanding of each individual’s disease etiology. Genetic studies using large databases are leading to more individualized risk prediction [10] for cognitive decline and dementia than was previously possible using only the established marker, *apolipoprotein E* genotype and have implicated novel targets for possible treatment, such as inflammatory pathways [11]. This kind of investigation may lead to novel and more sensitive biomarker screens to identify trial participants at an earlier stage of disease, when effective interventions may be more easily demonstrated.

However, a targeted approach will require substantial time and resources to develop, and even if it achieves some clinical success, it will be difficult to implement on a global scale. Costly screens and personalized treatments may be feasible in high-income countries (HICs) but may offer minimal relief to the growing proportion of people living with dementia in low- and middle-income countries (LMICs). In the meantime, a more accessible approach can be to address the lifestyle and environmental factors that contribute to dementia risk. Says Brayne, “Trials and drug development have to be done in parallel with societal thinking about cost and implications of taking this approach versus life-course optimization of brain health.” Epidemiological evidence suggests that late-life resilience to cognitive decline may be influenced by factors such as physical exercise, social and intellectual stimulation, and socioeconomic circumstances decades earlier as well as late in life [12–14]. Moreover, evidence is accumulating that long-term effects of traumatic brain injury [15,16], including concussive and potentially even subconcussive events, may represent an avenue for prevention, not only for professional athletes or soldiers exposed to head injury into their adulthood but also for people who play contact sports as children or adolescents.

Even an optimistic forecast shows little current likelihood of disease-modifying treatment for those already affected by dementia and few clear approaches to prevention for older populations at risk of progressive cognitive decline. For these people and those who care for them, the pressing concern is not eliminating risk or developing treatments for the next generation but how to manage established disease and prepare long-term care plans for the time when dementia care becomes too much to handle without help. As with targeted therapies, care options differ across socioeconomic strata. For people in care homes, new person-centered psychosocial interventions [17,18] may provide benefit to quality of life and decreased reliance on medications. However, for most in LMICs (and many people in HICs), care is delivered by overstretched institutions or directly by families, which may both be lacking the capacity to provide the personal attention and high-level care that people with dementia require and deserve. This is the current crisis: the human and economic costs of dementia each year far outweigh the research investments that any pharmaceutical company is willing to stake on effective interventions. The response will no doubt differ among countries and depend on local resources and disease burden, but pending a more effective research agenda, it will surely demand the best forms of humane, supportive care that our family ties, social consciences, and economic systems can provide.

## Abbreviations

AD: Alzheimer disease
HIC: high-income country
LMIC: low- and middle-income country
NFT: neurofibrillary tangle
